# [Retracted] MicroRNA‑320 targets mitogen‑activated protein kinase 1 to inhibit cell proliferation and invasion in epithelial ovarian cancer

**DOI:** 10.3892/mmr.2024.13169

**Published:** 2024-01-22

**Authors:** Yongqian Xu, Jian Hu, Chunxia Zhang, Yuanyuan Liu

Mol Med Rep 16: 8530–8536, 2017; DOI: 10.3892/mmr.2017.7664

Following the publication of this paper, it was drawn to the Editor's attention by a concerned reader that certain of the Transwell cell invasion assay data shown in Fig. 5C on p. 8534 were strikingly similar to data that had already been published in different form in different articles written by different authors at different research institutes, or were submitted for publication at around the same time (several of which have now been retracted).

Owing to the fact that some of the data in the above article had already been published prior to its submission to *Molecular Medicine Reports*, the Editor has decided that this paper should be retracted from the Journal. The authors were asked for an explanation to account for these concerns, but the Editorial Office did not receive a reply. The Editor apologizes to the readership for any inconvenience caused.

